# 3D Ear Identification Based on Sparse Representation

**DOI:** 10.1371/journal.pone.0095506

**Published:** 2014-04-16

**Authors:** Lin Zhang, Zhixuan Ding, Hongyu Li, Ying Shen

**Affiliations:** School of Software Engineering, Tongji University, Shanghai, China; University of Maryland, College Park, United States of America

## Abstract

Biometrics based personal authentication is an effective way for automatically recognizing, with a high confidence, a person’s identity. Recently, 3D ear shape has attracted tremendous interests in research field due to its richness of feature and ease of acquisition. However, the existing ICP (Iterative Closet Point)-based 3D ear matching methods prevalent in the literature are not quite efficient to cope with the one-to-many identification case. In this paper, we aim to fill this gap by proposing a novel effective fully automatic 3D ear identification system. We at first propose an accurate and efficient template-based ear detection method. By utilizing such a method, the extracted ear regions are represented in a common canonical coordinate system determined by the ear contour template, which facilitates much the following stages of feature extraction and classification. For each extracted 3D ear, a feature vector is generated as its representation by making use of a PCA-based local feature descriptor. At the stage of classification, we resort to the sparse representation based classification approach, which actually solves an l_1_-minimization problem. To the best of our knowledge, this is the first work introducing the sparse representation framework into the field of 3D ear identification. Extensive experiments conducted on a benchmark dataset corroborate the effectiveness and efficiency of the proposed approach. The associated Matlab source code and the evaluation results have been made publicly online available at http://sse.tongji.edu.cn/linzhang/ear/srcear/srcear.htm.

## Introduction

In modern society, with the development of hardware and the awareness of security among people, recognizing the identity of a person with high confidence has become a key issue and a topic of intense study [1]. Conventional card based, or user name and password based, authentication systems have shown unreliability and inconvenience to some extent. Instead, biometrics based methods, which use unique physical or behavioral characteristics of human beings, are drawing increasing attention in both academic research and industrial applications because of their high accuracy and robustness in the modern e-world. With the rapid development of computing techniques, in the past several decades or so, researchers have exhaustively investigated a number of different biometric identifiers, including fingerprint, face, iris, palmprint, hand geometry, voice, gait, etc [2].

Compared with classical biometric identifiers such as fingerprint [3] and face [4], the ear is relatively a new member in the biometrics family and has recently received some significant attention due to its non-intrusiveness and ease of data collection. As a biometric identifier, the ear is appealing and has some desirable properties such as universality, uniqueness and permanence [5], [6]. The ear has a rich structure and a distinct shape which remains unchanged from 8 to 70 years of age as determined by Iannarelli in a study of 10,000 ears [5]. As pointed out by Chang *et al.* [7], the recognition using 2D ear images has a comparable discriminative power compared with the recognition using 2D face images.

According to the types of input data, ear recognition problems can be classified as 2D, 3D, and multimodal 2D plus 3D. Most existing studies made use of only 2D profile images and the representative works belonging to this category can be found in [7]–[14]. Besides the traditional 2D ear sensing, there now also exists technologies to acquire 2D plus 3D ear data simultaneously. With the developing and popularizing of 3D sensor technology, there is a rising trend to use 3D sensor instead of traditional 2D camera in ear recognition research. Compared with 2D data, 3D ear data contains more information about ear shape and is not sensitive to illumination and occlusion. In [15], Yan and Bowyer found that ear matching based on 3D data could achieve a higher accuracy than that making use of the corresponding 2D images.

In this paper, we address the problem of 3D ear identification. To this end, we propose a novel effective and efficient fully automatic 3D ear recognition approach based on the sparse representation framework [16]. The remainder of this paper will be arranged as follows. Related work and our contributions of this paper are described in Section 2. The proposed template-based ear detection approach is elaborated in Section 3. The feature extraction scheme and the classification approach are described in Section 4. Results of performance evaluations are presented in Section 5. Finally, Section 6 concludes the paper.

## Related Work and Our Contributions

### 1. 3D Ear Recognition

3D ear biometrics is relatively a new research area and there have been a few studies conducted. Some prominent and representative relevant works are briefly reviewed here. Chen and Bhanu are among the earliest scholars conducting research on 3D ear biometrics. In [17], they proposed a method for detecting the ear region from a profile range image. Their approach is a two-step system, including model template building and online detection. The model template is obtained by averaging the shape index histograms of multiple ear samples. The online detection process consists of four steps, step edge detection and thresholding, image dilation, connected-component labeling, and template matching. In [18], Chen and Bhanu proposed a shape-model based technique for locating ears in a side face range image. However, how to match two ears was not mentioned in [18]. In [19], Chen and Bhanu proposed a two-step iterative closest point (ICP) [20] based approach for 3D ear matching. In the first step, the helix of the test ear is detected and is coarsely aligned with the model ear helix by using ICP; in the second step, the ICP algorithm iteratively refines the transformation to bring model ears and the test ear into best alignment. The root mean square (RMS) distance is used as the matching error criterion. The shortcoming of the work [19] is that all the ear regions were extracted from profile images manually. In their later work [21], Chen and Bhanu developed a fully automatic 3D ear matching framework. In [21], they proposed two shape representations for 3D ear, namely, a local surface patch (LSP) representation and a helix/antihelix representation. Both of the shape representations are used to estimate the initial rigid transformation between a gallery-probe pair. A modified ICP algorithm is then used to iteratively refine the alignment. In [22], Yan and Bowyer conducted an experimental investigation of ear biometrics and they exploited several different approaches, including the eigen-ear method using 2D intensity images as input, principal component analysis (PCA) applied to range images, Hausdorff matching of depth edge images derived from range images, and ICP-based matching of the 3D data. In their later work [23], Yan and Bowyer proposed a fully automatic 3D ear recognition system, in which for ear region detection they tried to locate the ear pit and then used the active contour algorithm [24] to extract the ear contour. With respect to the strategy for matching two ears, they again resorted to ICP. In [25], Passalis *et al.* proposed a generic annotated ear model (AEM) to register and fit each 3D ear and then a compact biometric signature was extracted containing 3D information. In their later paper [26], they extended their work by developing a semi-automatic multi-modal 3D face and 3D ear recognition system. Such a system processes each modality separately and the final decision is determined based on the weighted average of the two similarity measures from the face and the ear modalities. In [27], Cadavid and Abdel-Mottaleb proposed an approach for 3D ear biometrics using video sequences. For each subject, a 3D ear model is derived from a video clip and ICP is adopted for computing the matching distance between two ear models. In [28], Islam *et al*. adapted the face recognition framework developed in their previous work [29] and proposed a coarse-to-fine 3D ear recognition approach. In their method, ear regions are detected from 2D profile images by training an AdaBoost classifier and then the corresponding 3D ear data is extracted from the co-registered range image. For ear matching, they also adopted ICP. In their latest work [30], Islam *et al*. tried to combine the 3D face and the 3D ear to build a multimodal biometrics system. In [31], Liu explored a fast recognition mechanism based on local surface matching with ICP registration to solve the 3D ear recognition problem under low cost strip point cloud environment. For a more comprehensive recent review of the ear biometrics, readers can refer to [32].

### 2. Sparse Representation in 3D Biometrics

It has been found that natural images can be sparsely coded by structural primitives [33] and in recent years sparse coding or sparse representation has been successfully applied to a variety of problems in computer vision and image analysis, including image denoising [34], image restoration [35], [36], object classification [16], [37], [38], visual saliency [39], and blind image quality assessment [40]. The great success of sparse representation can be partially attributed to the progress of *l*_0_-norm and *l*_1_-norm minimization techniques [41]–[46].

In [16], Wright *et al*. made use of the sparse representation based classification (SRC) framework to address the face recognition problem and achieved impressive performances. With SRC, a query face image is first sparsely coded over the gallery images and then the classification is performed by checking which class yields the least coding error. Motivated by the great success of Wright *et al*.’s work, some other researchers have tried to apply SRC to other kinds of classification problems and among these works there are a few relevant to 3D biometrics. A representative work specializing on applying SRC to 3D biometrics is [47]. In [47], Li *et al*. proposed a 3D face recognition approach based on SRC. In their work, for each 3D face, a feature vector comprising a set of low-level features, such as the curvature at the vertex, the area of each triangle, the length of each edge etc., is extracted and fed into the sparse representation based classifier for classification. With a similar idea as [47], there are other two works also focusing on SRC-based 3D face recognition [48], [49]. The difference among the works [47], [48] and [49] mainly lies in that they made use of different schemes for the feature extraction and selection.

### 3. Motivations and Contributions

From the aforementioned introduction and analysis, it can be seen that most existing 3D ear matching methods are based on ICP or its variants. While ICP is a feasible 3D matching approach for the one-to-one verification case, it is not quite appropriate for the one-to-many identification case. If there are multiple samples for each subject in the gallery set, to figure out the identity of a given test sample using an ICP-based matching method, it would be necessary to match the test sample to all the samples in the gallery set one-by-one. With the number of gallery samples rising, the performance of ICP-based methods will markedly drop down. Actually, the task of identification is essentially to find out a single class containing samples most similar to the input test sample out of the entire gallery set. To solve such a one-to-many identification problem, Wright *et al.* [16] have demonstrated that SRC is an effective and efficient tool. In addition, researchers have also shown the feasibility of making use of SRC to solve the 3D face recognition problem [47]–[49]. However, to the best of our knowledge, there is no work so far reported to apply SRC to address the 3D ear recognition problem.

**Figure 1:**
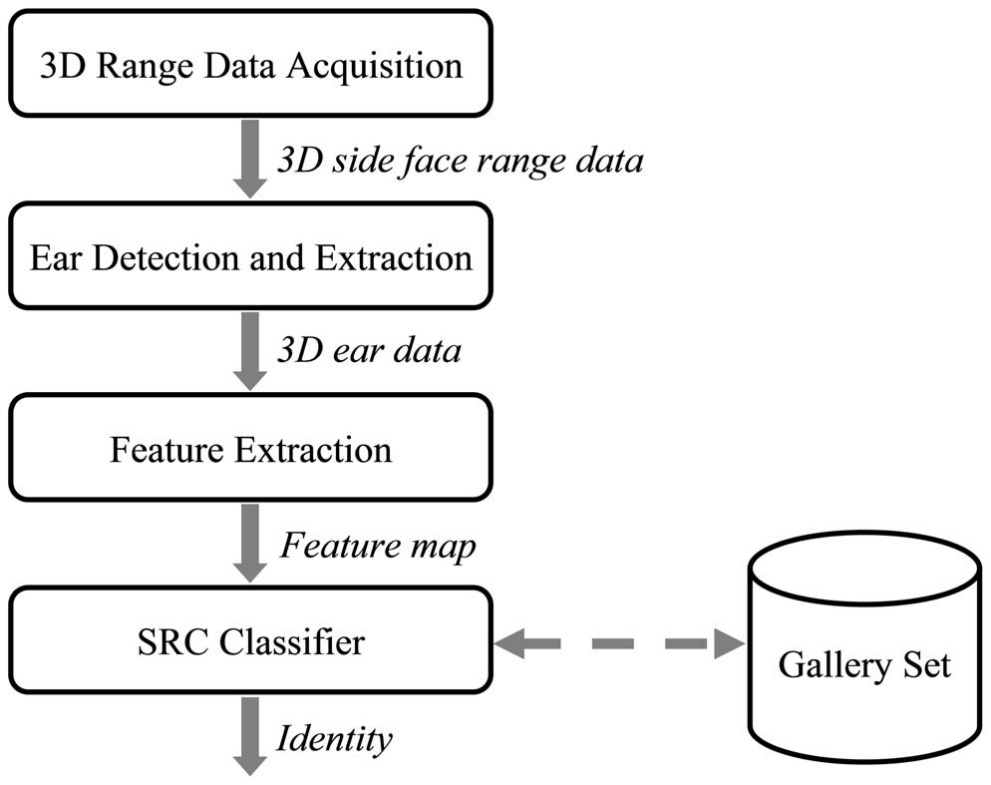
The general flowchart of our proposed 3D ear identification approach.

Based on these considerations, in this paper, we propose a novel fully automatic 3D ear identification approach based on sparse representation, whose general architecture is shown in Fig. 1. From Fig. 1, it can be seen that our approach mainly consists of three components, ear detection and extraction, feature extraction, and SRC-based classification. To match the SRC classifier used at the classification stage, for ear detection, we propose a template based scheme which is robust to pose change. Since with such an ear detection scheme the extracted ear region will be aligned to the template ear contour model, all the extracted ears consequently reside in a common canonical coordinate system. Therefore, at the identification stage, we do not need to register the test ear sample to the gallery samples, which is crucial for the usage of SRC. For feature extraction, we propose an effective PCA-based local descriptor for the local 3D range data, which is highly inspired by Mian *et al*.’s salient work [50]. Feature vectors are extracted from ear samples in the gallery set and they form an overcomplete dictionary **A**. When a new test ear sample comes, its feature vector **y** will be extracted and then its identity can be figured out by using the SRC which codes **y** over the dictionary **A**. Our approach takes the 3D range image as the only input and no extra color image is required. The performance of the proposed approach is evaluated on the benchmark dataset and is compared with the ICP based method. Efficacy and efficiency of our approach are corroborated by the experimental results.

## Template-based Ear Detection

In this section, the proposed template-based ear detection method will be presented. Actually, most of the existing ear detection or ear region extraction approaches use only 2D profile images. However, when the ear data is acquire by 3D sensors, sometimes the corresponding 2D color images cannot be gained or have occlusion problems. Thus, in our paper we present an automatic ear detection approach totally based on 3D range data. In our work, however, the role of the ear detection is not only to locate and extract the ear region out of the input 3D ear data but also to align the extracted ear region to a template ear contour model. In this way, all the extracted ears are represented in a common canonical coordinate system. In our system for each ear, the input 3D ear data is just a 640×480 side face range scan. Since there are pixels which fail to record the corresponding vertices for a range scan, a binary image (usually provided along with the 3D range data) is utilized to indicate whether a pixel contains a vertex.

### 1. Ear Pit Detection

The binary image actually is a mask, each pixel of which indicates whether the corresponding pixel in the range scan contains a valid vertex. In order to locate the ear pit, we first need to locate the nose tip, which will greatly narrow down the searching range for the ear pit in the following process. Identifying the location of the nose tip is accomplished in the image space of the binary mask, which comprises the following two steps,

- Record the *X* value along each row at which we first encounter a white pixel in the binary image and find the mean value *X_mean_* of *X* values.
- Record the *Y* values of those rows whose starting “white” pixels have *X* values smaller than *X_mean_* and greater than *X_mean_* - 100. And we denote the mean of these *Y* values by *Y_mean_*. Within a 60 pixel range above and below of the *Y_mean_*, the valid point with minimum *X* value is the nose tip and its position is represented by (*X_NoseTip_*, *Y_NoseTip_*).

**Figure 2:**
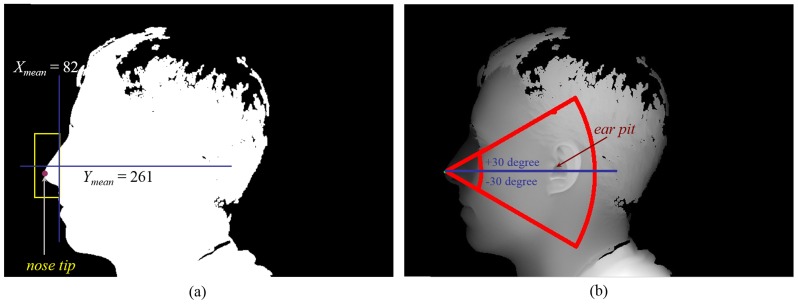
Illustration for the proposed ear pit detection scheme. (a) The nose tip is first located in the binary mask. (b) When the nose tip is detected, we can further locate the ear pit within a sector associated to the nose tip.

We use the *Z* value in the range scan as the intensity for each pixel to generate a 2D intensity image, and the further locating of ear pit is performed in such an image. Based on the location of the nose tip, we can generate a corresponding sector which the ear pit should fall in. Such a sector can narrow down the searching range for identifying the ear pit. With the point (*X_NoseTip_*, *Y_NoseTip_*) as the center, we define this sector as a fan region spanning +/−30 degrees from the horizontal. In this sector, we reject pixels of which the corresponding vertices have a distance larger than 16 cm or smaller than 4 cm to the nose tip. To identify the ear pit, we propose a simple yet effective method. We assume that the ear pit should be the point of which the *Z* value is the lowest within a circular range. Following this rule, we pre-generate a random sample pattern which consists of 150 points within a circle whose radius is 30-pixels. For each point falling in the sector, we sample the points in its neighborhood via the pre-generated sample pattern. The points with the lowest depth values in their local neighborhoods are valid candidates for the ear pit. By observing the distribution of these candidates, we found that the true ear pit must lie on a cluster which contains several candidates. Hence, we remove the isolated candidates having no other candidates around, which could be caused by noise or hair interference. From the remaining candidates, we regard the one with the smallest depth value as the ear pit. Procedures for the ear pit detection are illustrated by using an example in Fig. 2.

### 2. Ear Contour Alignment and Ear Region Extraction

In order to partially solve the pose change problem and to make the extracted ear regions reside in a common canonical coordinate system, we adopt the ICP algorithm to align the ear contour of the sample being processed to an ear contour template created offline manually. Such an idea is inspired by the work [21]; however, there are some differences. At first, in our case, since the ear pit has already been detected, the computational cost of ICP-based contour matching could be reduced by aligning the ear pits first. Secondly, in our case, ICP matching is performed in the 2D image space, which is much faster than the one working in the 3D space.

**Figure 3:**
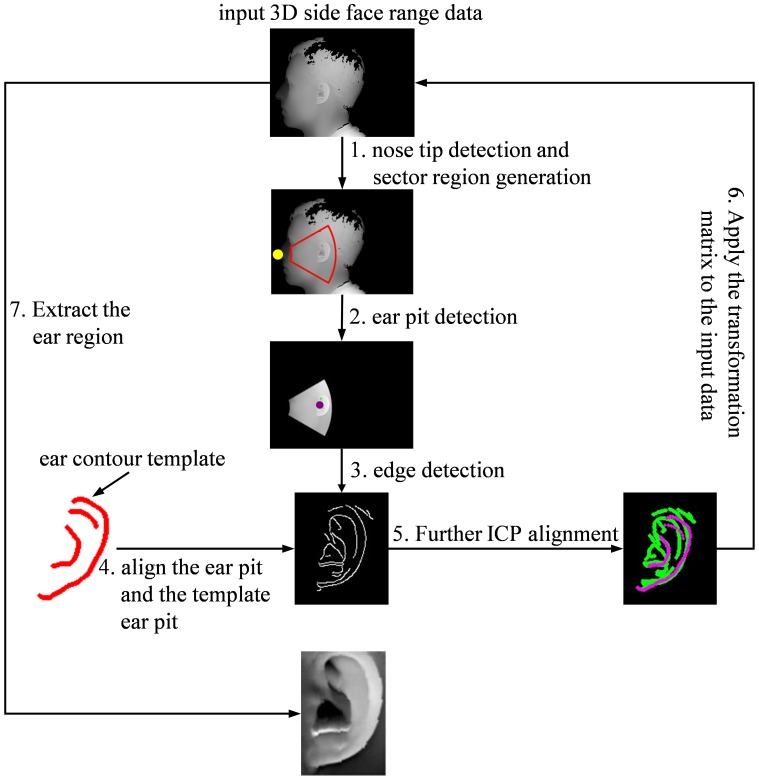
Flowchart of the proposed ear detection scheme.

We built an ear contour template by manually selecting the ear pit point, 30 helix points, and 10 antihelix points from one instance of samples from UND-J2 ear database [51]. The ear contour template finally generated is shown in Fig. 3. For each 3D side face image being processed, we at first extract the sector region containing the ear. Then, we apply a Canny edge detector on the extracted sector of the depth image and we can consequently get an edge map which can be regarded as the contour of the ear. After that, we register the obtained edge map to the ear contour template roughly by aligning the detected ear pit to the template ear pit. Then, an ICP algorithm is applied in the image space to refine the alignment further. After that, we transform the original input 3D range data into the coordinate system defined by the ear contour template by using the translation and rotation matrices obtained in the ICP alignment. Finally, a pre-defined rectangular region is extracted from the transformed range data and it is taken as the resulting ear region.

By using the template-based contour alignment, the final extracted 3D ear region is robust to ear pose change and there is little difference for the ears extracted from the same person. In addition, with such a scheme, all the extracted ear regions are represented in the same canonical coordinate system defined by the ear contour template, which facilitates the following feature extraction and classification. Fig. 3 illustrates the main steps involved in our proposed ear detection scheme.

## Ear Identification based on Sparse Representation

In this section, we will present our SRC-based ear identification approach in detail. At first, how to extract features from 3D ear range data will be introduced. Then, details for classification will be presented.

### 1. Ear Feature Extraction

In order to make use of the SRC for classification, we need to map the extracted 3D ear data into a feature vector of a fixed length. The commonly used features for 3D shapes, like Li *et al.* adopted in [47], are point curvature, triangle area, and so on. But the difficulty is to build one-to-one correspondences between two 3D shapes. In our case, however, since we have aligned all 3D ear data to a canonical coordinate system defined by the ear contour template in the ear detection step, the one-to-one correspondences have already been built automatically and the feature extraction could be directly applied to the extracted 3D ear data.

Instead of using traditional features like curvatures, we proposed a local PCA-based feature descriptor greatly inspired by Mian *et al*.’s work [50]. The extracted 3D ear could be represented as a point cloud: **E** = {[*x_i_, y_i_, z_i_*]*^T^*, where *i = *1,…, *n*}. For each point *p_i_* in **E**, let **L** = {[*x_j_, y_j_, z_j_*]*^T^*, where *j* = 1,…, *n_i_* } be the points in a region cropped by a sphere of radius *r* centered at the point *p_i_*. Given the dataset **L**, we can calculate its mean vector **m** and its covariance matrix **C**,
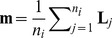
(1)

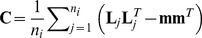
(2)where **L** *_j_* represents the coordinate of the *j* ^th^ point in the point set **L**. Then, an eigen-value decomposition is performed on the covariance matrix **C** and we can get a matrix **V** comprising eigenvectors and a diagonal matrix **D** of eigenvalues of **C**,



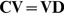
(3)Then, we can map point set **L** to **L**′ by,
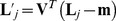
(4)


Let **L**′ *x* and **L**′ *y* be the projections of points **L** on its first and second principal components, respectively (these two principal components correspond to the first two largest eigenvalues). The feature value at point *p_i_* could be defined as,
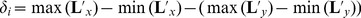
(5)


**Figure 4:**
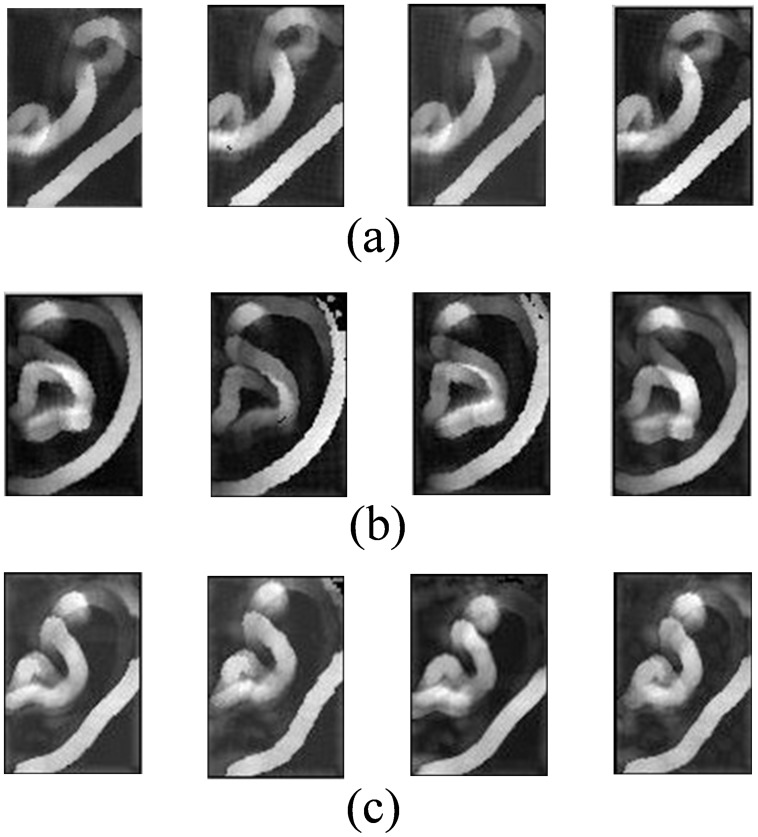
Feature samples computed by using the proposed feature extraction method. (a) Feature maps from different samples of ear A. (b) Feature maps from different samples of ear B. (a) Feature maps from different samples of ear C.

According to Mian *et al*. [50], *δ_i_* actually represents the difference between the lengths of the first two principal axes of the local region around the point *p_i_* and it will be zero if the point cloud **L** is planar or spherical. However, if there is unsymmetrical variation in **L**, then *δ_i_* will have a non-zero value proportional to the variation. Such a PCA-based local feature descriptor was originally proposed for key-points detection [50]. In [50], Mian *et al*. regard the points with high *δ_i_* values as key points. In our case, however, we simply utilize *δ_i_* as a local feature descriptor. After computing {*δ_i_*} for all the points {*p_i_*} in the ear region, we can obtain a feature map. In order to be used by the following classification stage, the feature map needs to be reformulated as a column vector. Fig. 4 demonstrates some examples of the calculated feature maps, shown in image format. The three rows of Fig. 4 correspond to three different ears while for each row, the four feature maps are computed from four different samples collected belonging to the same ear. From Fig. 4, we can have the following findings. At first, the proposed feature can reflect anatomical information of the ear very well. Secondly, by using the proposed method, feature maps derived from the different samples of the same ear look quite similar to each other while the ones computed from different ears are apparently different.

The dimension of the feature vectors obtained with the above scheme is quite high and it is not effective to directly feed them into the SRC for classification. Therefore, a further dimension reduction operation is necessary and to this end, we adopt the random projection approach introduced by Wright *et al*. in [16], which is quite simple yet effective.

### 2. Ear Recognition via SRC

Given an ear gallery set, we compute a feature vector **v** from each ear in gallery and form them to a dictionary matrix **A** *= *[**v**_11_,*…,* **v**_1*k*,_ **v**_21_,*…,* **v**_2*k*_ *,…,* **v** *_n_*_1_,*…,* **v** *_nk_*]
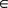
IR*^m^* ^×*nk*^, *m* here represents the feature dimension, *n* represents the number of ears, and *k* represents the number of samples for each ear in the gallery.

Given a query ear sample, denote by **y** its feature vector. The recognition problem can be viewed as solving the following over-completed linear equation,
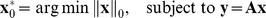
(6)
x* 0 here is a *l*_0_-minimization solution for this equation. However, the problem of identifying the sparsest solution of an underdetermined system of linear equations is NP-hard and difficult even to approximate. Fortunately, recent development in optimization theories reveals that if the solution x* 0 sought is sparse enough, it can be well approximated by the solution of the following *l*_1_-minimization problem [41],



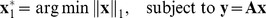
(7)With x* 1, we can compute the reconstruction residual when using the samples of the ear class *i* to approximate the test sample **y** as,
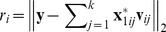
(8)where x* 1*ij* represents the (*ij*)^th^ coefficient of the solution vector x* 1. We then can classify **y** based on these reconstruction residuals by assigning it to the ear class that minimizes the reconstruction residual.

To better handle the noise and corruption problem, we used an extensional sparse representation by substituting **B** = [**A**, **I**] for the original **A** where **I** is the identity matrix. Therefore, the final sparse representation algorithm we adopt is,
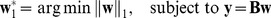
(9)


We extract x* 1 by decomposing w* 1 as w* 1 = [x* 1, **e**] and the construction residual calculation should also be substituted by,
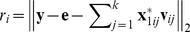
(10)


We can easily recognize any given query ear by solving the above *l*_1_-minimization problem in one matching step. For solving the *l*_1_-minimization problem, several prominent algorithms have been developed in the past few years, including Homotopy [42], FISTA [43], DALM [44], SpaRSA [45], *l*_1__*ls* [46], etc. In this paper, we adopt DALM algorithm [44] as the *l*_1_-minimization solver, which has been proved fast and accurate.

**Figure 5:**
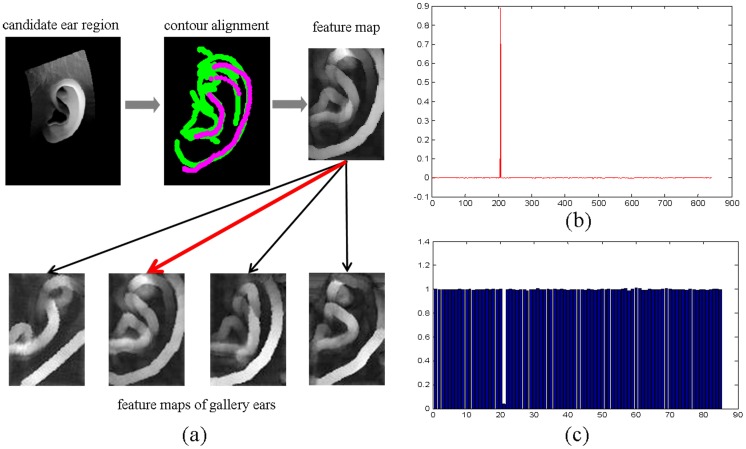
Illustration for the key steps of SRC-based 3D ear identification. (a) Illustration for the process of the proposed SRC based ear identification approach. (b) The values of the sparse coefficients recovered by SRC. (c) Reconstruction residuals obtained by using different ear classes to reconstruct the input test sample.

In Fig. 5(a), we use an example to demonstrate the process of the proposed SRC-based ear identification approach. After the ear region is extracted and aligned with the ear contour template, its feature map is extracted. Then, such a feature map is stacked into a feature vector and fed into the SRC for classification. From Fig. 5(b), we can see that if the identity of the test sample exists in the gallery, the solved solution is very sparse and usually, the largest coefficient corresponds to the gallery sample most similar to the test sample. Fig. 5(c) plots the reconstruction residuals obtained by using different gallery classes to reconstruct the test sample. The least reconstruction residual usually occurs with the class having samples most similar to the test sample and thus the reconstruction residual can be an indication of the potential identity of the test sample.

## Experimental Results and Discussions

**Figure 6:**
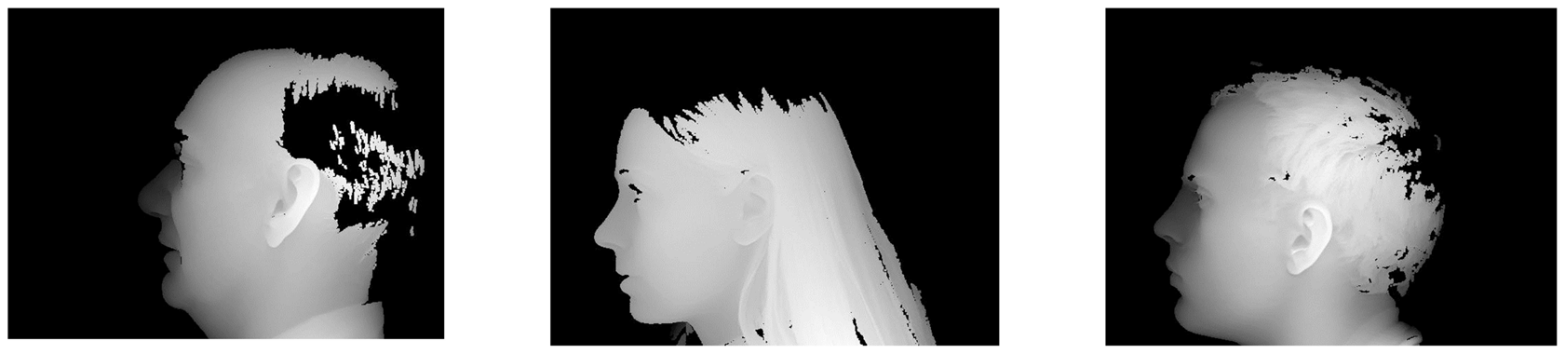
Samples of 3D side face range data in UND-J2 database.

In this section, we will present the evaluation results of the proposed method. The database we used in our experiment is UND collection J2 dataset [51]. The UND-J2 dataset is currently the largest 3D ear dataset which consists of 2436 side face 3D scan from 415 different persons. Several samples are shown in Fig. 6. Each 3D ear data is a 640×480 3D scan.

### 1. Evaluation of the Ear Detection Performance

To validate our ear detection algorithm, we manually marked ear pits for the whole UND-J2 dataset. In our experiment, we run the experiment on all the 2436 ears of UND-J2. For each 3D ear, we compared the automatic detected ear pit location (*X*_auto_earpit_, *Y*_auto_earpit_) with the ground-truth ear pit location (*X*_gt_earpit_, *Y*_gt_earpit_) and if the Euclidean distance of two positions were larger than 16 pixels, we regarded the ear detection for this ear as failure.

Under these experimental settings, our algorithm could achieve a 90.87% detection rate which is much higher than 85% reported in [28] by using the method proposed in [23] on the same dataset.

### 2. Evaluation of the Identification Performance

Although there are 415 subjects in UND-J2 database, most subjects have only 2 samples. Since the recognition based on sparse representation needs sufficient samples for each class in the gallery [16], we cannot run our experiment on the whole database.

In our experiment, we selected three subsets from UND-J2 database. The first subset contained 185 ears of UND-J2, each of which had more than 5 samples and the second subset contained 127 ears, each of which had more than 7 samples and the third subset contained 85 ears, each of which had more than 10 samples. For subset1, we randomly selected 5 samples from each ear to form the gallery and the rest of ears were formed to the test set. So the gallery size for subset1 was 925, and the test set size was 885. For subset2, we randomly selected 7 samples from each ear to form the gallery and the rest of ears were formed to the test set. Thus, the gallery size for subset2 was 889, and the test set size was 588. For subset3, we randomly selected 10 samples from each ear to form the gallery and the rest of ears were formed to the test set. Consequently, the gallery size for subset3 was 850, and the test set size was 291.

We tested our ear recognition algorithm on those three different subsets respectively and we also evaluated the performance of ICP under the same condition. Since there were multiple samples for each ear class in the gallery, we used ICP to match a query ear with all the samples for each ear class and regarded the minimum matching error as the matching error for that ear class. Finally, we took the label of the ear class generating the minimum matching error as the identity for that query ear. Table 1 lists the rank-1 recognition rates achieved by using our algorithm and ICP, where *M* stands for the number of samples for each ear class in the gallery. Table 2 lists the time cost consumed by one identification operation, where *N* stands for the gallery size.

**Table 1:** Rank-1 recognition rate.

	M = 5	M = 7	M = 10
ICP	83.83%	89.64%	94.09%
**Our Algorithm**	**87.79%**	**91.53%**	**95.23%**

**Table 2:** Time cost for 1 identification operation (seconds).

	N = 850	N = 889	N = 925
ICP	127.45	131.61	144.31
**Our Algorithm**	**0.041**	**0.044**	**0.047**

### 3. Further Discussions

From experimental results shown in Table 1, it can be seen that our proposed method performs better than ICP in terms of rank-1 recognition rate.

The greatest advantage of our algorithm over ICP is that it has a low time cost. Table 2 compares the computational time cost for one ear query process. To recognize the identity for one query ear, the ICP based algorithm has to compare the query ear to all the gallery ears and each comparison needs an ICP alignment for the two ear shapes. Without a previous rejection process, the whole recognition process will cost a lot of time. Different from ICP, the recognition process based on sparse representation just solves an *l*_1_-minimization problem based on pre-calculated features, so it is much faster than ICP based approaches. With gallery size rising, the computational time of ICP approach will hugely rise. However, the computational time of sparse representation based on DALM algorithm changes little with the enlargement of the gallery size, which can be reflected from the results listed in Table 2.

## Conclusions

In this paper, we proposed a novel 3D ear identification approach. In order to make use of the sparse representation framework for identification, we proposed a novel template-based ear detection method. By using this method, extracted ear regions are in a common canonical coordinate system defined by the ear contour template, which highly facilitates the following feature extraction and recognition steps. Compared with the classical ICP-based ear matching methods which will match the test sample to all the gallery samples one-by-one to determine its identity, the proposed SRC-based method is more efficient. Experiments were conducted on UND-J2 3D ear database and the results indicate that the proposed method could achieve high ear detection rate, high identification accuracy, and low computational cost.
